# Gilles de La Tourette syndrome: the contribution of Guinon

**DOI:** 10.1055/s-0045-1812298

**Published:** 2025-10-27

**Authors:** Hélio Afonso Ghizoni Teive, Francisco M. Branco Germiniani, Marcus Vinícius Della Coletta, Carlos Henrique Ferreira Camargo, Léo Coutinho, Olivier Walusinski

**Affiliations:** 1 Universidade Federal do Paraná, Departamento de Clínica Médica, Serviço de Neurologia, Curitiba PR, Brazil.; 2 Universidade Federal do Paraná, Programa de Pós-Graduação em Medicina Interna, Disciplina de Doenças Neurodegenerativas, Curitiba PR, Brazil.; 3 Universidade Federal do Amazonas, Hospital Getúlio Vargas, Manaus AM, Brazil.; 4 Private practice, Brou, France.

**Keywords:** Neurology, Movement Disorders, Tics, Tourette Syndrome, History, 19th Century

## Abstract

We review the contributions of Georges Guinon, an eminent pupil of Professor Jean-Martin Charcot, to the clinical description of Gilles de La Tourette syndrome, including phenomenological definitions and the occurrence of psychiatric findings such as obsessive-compulsive behavior.

## INTRODUCTION

Professor Jean-Martin Charcot's (1825–1893) contribution to the description of various neurological diseases is widely known. 1 2 3 In the field of movement disorders, particularly the study of tics and the definition of Gilles de La Tourette's syndrome (GTS), Charcot and his pupils presented invaluable contributions. 2 4 5 6 7 Although the works of Charcot and Gilles de La Tourette for the description of the syndrome are well known, one of Charcot's pupils, Georges Guinon (1859-1932), also presented an instrumental contribution to the understanding of tic disorders.

The present study reviews the contributions of Georges Guinon in describing the syndrome now defined as GTS.

## GEORGES GILLES DE LA TOURETTE (1857–1904)

George Gilles de La Tourette was born on October 30, 1857, in Saint-Gervais-les-Trois-Clochers, France. He died on May 22, 1904, in Lausanne, Switzerland. 4 5 6 7 8 9 10 Gilles de La Tourette started his activities as a clinician at the Salpêtrière Hospital, in 1884. He was later promoted to the post of Charcot's registrar, and finally to *Chief de Clinique* , in 1887 to 1888. 5 6 7 9 10 Gilles de La Tourette's academic career will always be remembered for his publications in the area of hysteria and hypnotism, as well as forensic medicine and movement disorders. 5 6 7 9 10 11 Nevertheless, his most important work was the first description in 1885 of a new disorder which Charcot would later name GTS. 5 6 7 9 10 11 12 13 14 15 While Gilles de La Tourette received the accolades, his description was incomplete and was further refined in works by Charcot himself and Georges Guinon ( Table 1 ). 4 5 6 9 11 13 14 15 16 17 18

**Table 1:** Contributions of Charcot, Gilles de La Tourette, and Guinon in the clinical definition of Gilles de La Tourette's syndrome (GTS)

Contribution/Author	Jean-Martin Charcot	Georges Gilles de La Tourette	Georges Guinon
The original idea for research	**+**	**-**	**-**
The first publication of GTS	−	+	−
Criticism to the title of GTS paper - “incoordenation motrice”	+	−	+
Obsessive-Compulsive Disorder: “idées fixes”:	+	−	−
“Folie du porquois”	+	−	+
“Folie du doute avec delirie du touches”	+	−	+
“Arithmomanie”	+	−	+
“Onomatomanie”	+	−	+
Tics temporarily inhibited	+	−	+
Waxing and waning symptoms	+	−	+
Heredity of GTS	+	−	−
Hysteria associated	−	−	+
Chorea in the differential diagnosis	+	+	+
Jumping French of Maine, Latah, and Myriachit	−	+	−
Echokinesis: Echopraxia and echolalia	+	+	+
Coprolalia	+	+	+

## GEORGES GUINON (1859–1932)

**Figure 1:**
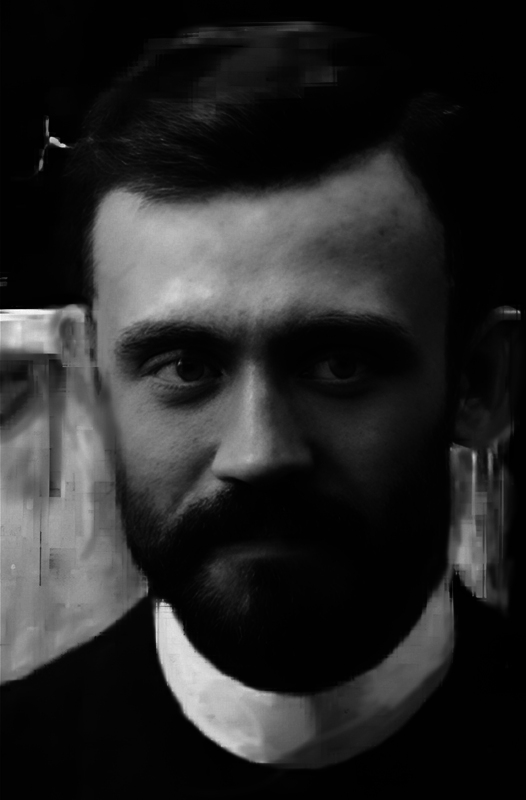
**Georges Guinon (1859–1932).** (Extracted from Baillement.com, January 2, 2025).

Georges Guinon ( Figure 1 ) was born in 1859 and died suddenly in Paris in 1932. 9 10 He became one of Charcot's 33 interns in 1885. 9 In that year, Charcot assessed Guinon's medical records as excellent (“ *interne très instruit, très dévoué”).* Later, around 1889 and 1890, Guinon succeeded Gilles de La Tourette as *Chief de Clinique* . Guinon was the fourth and last neurologist to work with Charcot in his private clinic. 9 10 He had great affection for and was very devoted to Charcot and wrote the famous paper “Charcot Intime.” 1 2 9 10 16 In 1889, Guinon presented his thesis, entitled “Les agents provocateurs de l'hysterie”, to a committee presided by Charcot. Subsequently, Guinon contributed significantly to other publications, such as “Leçons sur les maladies du système nerveux faites à La Salpêtrière.” 9 10 His interest in tics led Guinon to publish on the subject, demonstrating concepts different from those defined by Gilles de la Tourette. 9 10

## DEFINING GILLES DE LA TOURETTE SYNDROME: THE CONTRIBUTION OF GUINON

Guinon contributed significantly to the definition of GTS and published, in 1886, a case series with patients followed up at Charcot's service presenting clinical features of GTS. 19 20

**Figure 2:**
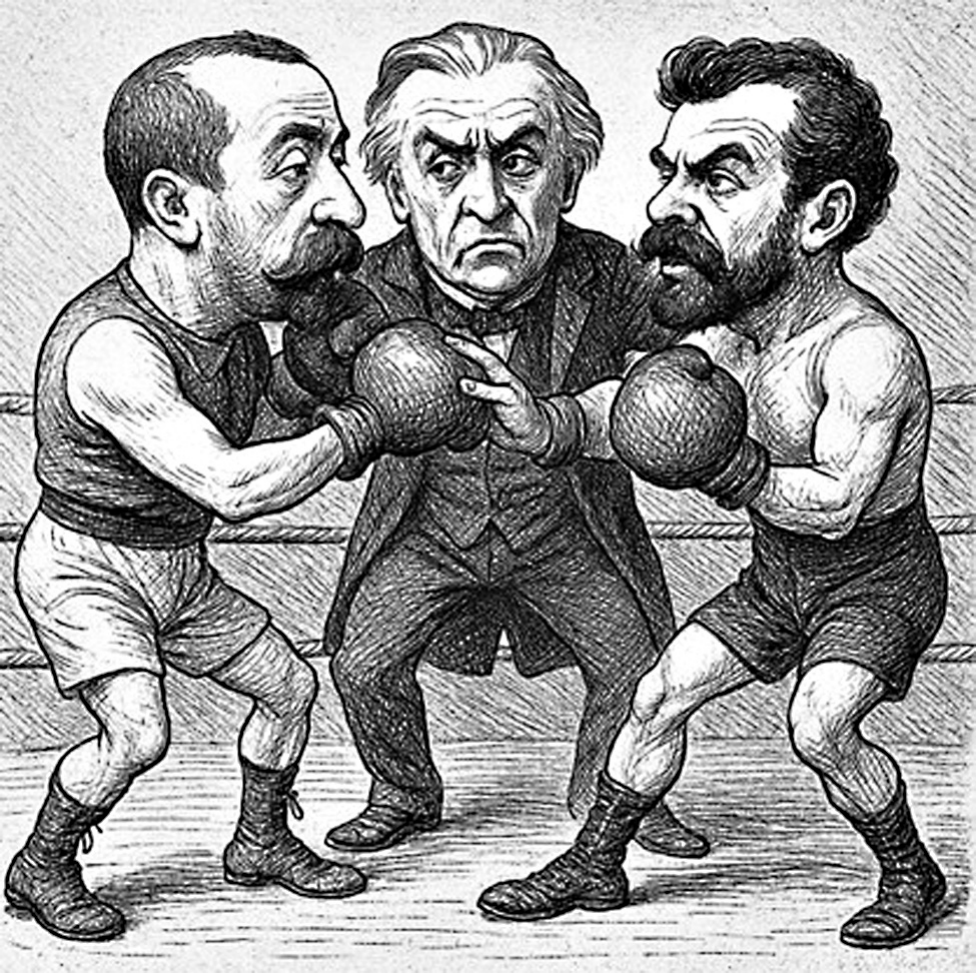
Guinon and Tourette, both under Charcot's tutelage, disagreed over the existence of motor incoordination in Gilles de La Tourette's syndrome (GTS) and the adequate terminology to name the disease, with Guinon preferring the term “La maladie des tics convulsifs”.

He disagreed with the terminology used by Gilles de La Tourette, “ *incoordination motrice* .” For Guinon, tics would be phenomenologically defined as involuntary movements, but they remain as coordinated movements, and the lack of motor coordination was not a feature of the disease. In addition to that, Guinon suggested using the term “ *la maladie des tics convulsifs* ( Figure 2 ).” 9

Additionally, Guinon recalled the psychiatric findings that accompanied the tics disorder, such as the presence of “ *les idées fixes* ,” already described by Charcot, that are now classified as obsessive-compulsive behavior. 5 6 9 11 13

He mentioned the “ *folie du porquois* ”, the “ *folie du doute avec délire du toucher* ,” “ *l'arithommanie (manie du calcul)* ,” or “ *l'onomatomanie* ” (distressing search for the right word), also described by Charcot and Valentin Magnan (1835–1916):

Monsieur Charcot drew our attention to the existence in our patients of a series of psychological phenomena which we have not found noted in other observations of this kind and which must, if we are to believe the few cases which we have studied, be quite frequent in the serious cases of tic disorder. These are fixed ideas. In an absolute sense, considered apart from the disease we are concerned with, the fixed ideas constitute, we know, an important chapter of mental pathology. We are aware that they can be extremely varied, and just to give a few examples, we will mention the *folie du pourquoi* , in which patients are irresistibly drawn to ask the reasons for absolutely insignificant things; the *folie du doute* , with *delire du toucher* , which differs slightly from fixed ideas in that it leads to genuinely fantastic ideas; arithmomania or number mania, and onomatomania, as recently described by Messieurs Charcot and Magnan. 19 20

Another feature defined by Guinon was the possibility of temporary suppression of tics:

When a spasmodic movement occurs in these patients, the excitement which provokes it comes from the cells of the cortex centres. Every movement of this kind is, as are voluntary movements, preceded by a motor representation of the same movement. And this is so true that the patient knows, at the precise moment when he is on the point of carrying out his tic, that it is going to happen, since he can in certain cases stop its external manifestation as an act of will. But in the general way of things, the will has lost this inhibitive power: the movement happens anyway. 19 20

Finally, Guinon declared that “the malady of convulsive tics” was a form of hysteria, contradicting Charcot's ideas. 9 Despite Guinon's insistence on using the term “ *la maladie des tics convulsifs* ,” Charcot suggested keeping the eponym GTS as he originally proposed. 9

In conclusion, Guinon presented invaluable contributions to the description of GTS, establishing the phenomenological definition of the involuntary movements and defining the accompanying obsessive-compulsive behavior that characterize the syndrome. These descriptions still influence our understanding of GTS in modern days and are, therefore, worthy of praise.
